# Supplementary material for: HIVprotI: an integrated web based platform for prediction and design of HIV proteins inhibitors
Source: J Cheminform. 2018 Mar 9;10:12. doi: 10.1186/s13321-018-0266-y (PMC5845081; doi:10.1186/s13321-018-0266-y)
Supplement: Supplementary file 2 — Additional file 2. Source code of HIVProtI web server. [file 13321_2018_266_MOESM2_ESM.zip › HIVprotI_Source-code/hivprotI/weka-3-6-6/doc/weka/filters/package-frame.html]

weka.filters


weka.filters

Interfaces 
  
*Sourcable*
  
*StreamableFilter*
  
*SupervisedFilter*
  
*UnsupervisedFilter*

Classes 
  
AllFilter
  
CheckSource
  
Filter
  
MultiFilter
  
SimpleBatchFilter
  
SimpleFilter
  
SimpleStreamFilter
